# Knowledge, perception, and fear among the global population towards newly evoked variant Omicron (B.1.1.529)

**DOI:** 10.1371/journal.pone.0270761

**Published:** 2022-07-06

**Authors:** Ali Qureshi, Syed Azhar Syed Sulaiman, Narendar Kumar, Pir Abdul Ahad Aziz Qureshi

**Affiliations:** 1 Department of Clinical Pharmacy, School of Pharmaceutical Sciences, USM, Pulau Penang, Malaysia; 2 Faculty of Pharmacy, University of Sindh Jamshoro, Jamshoro, Pakistan; 3 Department of Radiology, Syed Abdullah Shah Institute of Medical Sciences, Sindh, Pakistan; King Abdulaziz University, SAUDI ARABIA

## Abstract

Severe acute respiratory syndrome coronavirus 2 (SARS-CoV-1), notoriously known as COVID-19, emerged in 2019 and was responsible for causing acute respiratory collapse. Moreover, in September 2020, new variant cases of severe acute respiratory syndrome coronavirus 2 were reported in the United Kingdom, with many patients and deaths. This study aimed to see knowledge, perception, and fear among the global population towards a new variant of severe acute respiratory syndrome coronavirus 2, known as Omicron (B.1.1.529). This online cross-sectional global study was conducted during the emergence of the B.1.1.529 variant, also known as the Omicron variant. The survey was carried out from 2^nd^ December 2021 to 3^rd^ January 2022. The descriptive analysis was presented as frequencies (N), percentages (%), and mean ± standard deviation (m ± SD). The association between dependent and categorical independent variables was determined using the Chi-square test (*x2*). Statistical analysis was performed by using SPSS version 23. Of 353 respondents, approximately 61% were females. One hundred fifty-four respondents were in the age group of 18–27 years. The average age was 31.53±10.3 (mean± SD). The majority of respondents (43.9%) were from Indonesia. The mean knowledge score about the Omicron variant was 3.18±1.14. Our study suggests that people have some knowledge about the new variant, Omicron (B.1.1.529). Besides, there was a significant association (p = 0.05) for the perception of the fatality rate of Omicron among the respondents from different countries. However, there is still an ample research gap in enlightening people about this infection (B.1.1.529).

## Introduction

Severe acute respiratory syndrome coronavirus 2, also known as COVID-19, erupted in 2019 and was associated with acute respiratory failure. The first case globally was reported in Wuhan city, China. The spread of this newly evoked infection was so abrupt that it was not getting stopped instead of restrictions. Later in March 2020, it was announced as a pandemic by the world health organization [1].

Moreover, in September 2020, new variant cases of Severe acute respiratory syndrome corona virus-2 were reported in the United Kingdom, with many patients and many deaths. The cases were stated up to fifty thousand daily until 2020. The first variant of COVID-19 was ever reported, known as B 1.1.7 (alpha variant). Another severe acute respiratory syndrome variant, coronavirus-2, rose from South Africa and was called B.1.351 (beta variant). Experts believe that the COVID-19 has undergone magnanimous mutations since it was evoked; however, many do not change the viral behavior [2].

In April 2021, B.1.617.2 (delta variant) was initially reported in India, with the highest count of approximately 16 million infected cases with over 2 lac deaths [3]. In the same year, variants were classified into two main groups named a variant of concern (VOC) and (a variant of interest (VOI), respectively [4,5]. However, it is still unclear to what degree these mutations change the antigenic nature of COVID-19, allowing variations to bypass protection given by vaccination. Furthermore, there is mounting evidence that modifications in the antigenic phenotype of Severe acute respiratory syndrome corona virus-2 are mingling and affecting immune recognition to the point where prompt action is required [6].

As per the World health organization, five variants of SARS-COV2 were declared as variants of concern [4] because the variant of concern spread more readily as compared to others [7]. The first is the alpha variant (B.1.1.7), which amplified Great Britain’s various parts in September 2020 [4,8]. The second is the beta variant (B.1.351) evoked from South Africa. Third and fourth is the Epsilon variant (B.1.427 and B.1.429) that emerged from the USA. The fifth one includes the gamma variant (P.1) that emerged from Brazil and Japan [4].

Various nations are experiencing major COVID-19 outbreaks and a sharp increase in mortality due to new variations. New variations can transmit more quickly and evade immune defenses. It is essential to understand the effectiveness of available vaccines against upcoming variants. Hence more research is required to explore re-infection, viral transmission, and escaping immune response [4,8,9]. The beta variant (B.1.351) had 4.6 times more affinity toward Angiotensin-converting enzyme-II (ACE-II) than the receptor-binding domain of the first Severe acute respiratory syndrome corona virus-2 [8,10]. Angiotensin-converting enzyme-II receptor is a crucial receptor to regulate the enzyme named Angiotensin-Converting Enzyme-II [11]. This shows that specific variants have more binding ability. As firstly detected in Brazil, the gamma variant (B.1.1.28.1) was involved in the second wave of COVID-19 in Brazil, with more potential for spread and lethality [8,12].

The world health organization (WHO) and the Center for Disease Control and Prevention (CDC) declared 11 variants as a variant of interest (VOI) [4]. It includes the Eta variant (B.1.525) evoked in the United Kingdom and Nigeria [4,13] and the kappa (B.1.617), and the delta variant (B.1.617.2 and B.1.617.3), which evoked from India. The world health organization entitled this variant of interest, but the Center for disease control and prevention entitled those variants as a variant of concern [14,15]. Delta variant was responsible for bringing the second wave to India and was identified in 92 other countries [16,17]. Other VOIs, include Theta (P3 or B.1.1.28.3), emerged from Japan, and its cases were found in the Philippines [18]. Another variant Zeta (P.2, B.1.1.28.2), appeared in Uruguay and various other regions of the world [19]. Similarly, the B.1.616 variant from France is also part of VOI class [20].

Initially, the Omicron (B.1.1.529) was detected in the specimen taken in Botswana on 11 November 2021. Later on, it was detected in South Africa on 14 November. On 30 November, Omicron was designated as a variant of concern by the World Health Organization (WHO). The first case in the United States of America was reported on 1 December 2021 among an international traveler from South Africa. However, another case was reported on 2 December 2021 among an individual without any traveling history. Omicron cases were reported in various countries, including Canada, Australia, Brazil, Japan, Norway, and the United Kingdom [7].

World health organization has entitled Omicron as the variant of concern due to its mutation that causes the chances of reoccurrence of illness again [21,22]. However, a South African study revealed no impact of vaccination on the prevalence of the omicron variant [23]. Moreover, the emergence of upcoming severe acute respiratory syndrome corona virus-2 may bring more chances of reoccurrence of COVID-19 infection [24]. Hence, globally it is vital to get well prepared for far coming variants [25]. Therefore, this study aimed to see knowledge, perception, and fear among the global population towards Omicron as a new variant of severe acute respiratory syndrome corona virus-2.

## Material and methods

### Study design, reliability and sample

This online cross-sectional study was conducted during the emergence of the B.1.1.529 variant, also known as the Omicron variant. The survey was carried out from 2nd December 2021 to 3rd January 2022. A self-structured questionnaire was devised on Google forms, and its link was circulated to the public through social media platforms (Whatsapp and Facebook messengers). The simple random sampling method was adopted in this study. A total of 385 responses were collected, with a response rate of 77%. Initially, a pilot study was performed on the first 25 responses using Cronbach’s Alpha coefficient test. The Cronbach’s alpha coefficient values for knowledge and perception were 0.69 and 0.75. The overall Cronbach’s Alpha coefficient value was 0.7. Finally, 353 respondents who agreed to participate were part of the study.

### Inclusion and exclusion criteria

Responses from those who agreed to participate were included in the study. All the responses from respondents aged 18 or above years were included. Responses to a pilot study were excluded. Respondents who refused to give consent to publish their responses were also excluded.

### Study instrument and measures

The questionnaire was designed in the English language. There was no restriction on age, gender, ethnicity/ country, marital status, and education level. A mandatory consent statement by respondents was entered at the start of the questionnaire. The questionnaire had various sections. The first section contained demographic information, vaccination status, and travel history. The second section included knowledge about COVID-19 and Omicron (B.1.1.529) variant. The third section was related to the perception of COVID-19, perception of variants of COVID-19, and perception of Omicron (B.1.1.529). The fourth section was about Fear of Omicron (B.1.1.529).

Demographic variables included; gender, age, marital status, Country, and education. Knowledge was measured on the basis of four questions purely related to Omicron (Table 4: K4, K5, K6, and K7). Each Omicron-related knowledge question was dichotomous and close-ended (Yes / No). Each positive reply (Yes) was assigned 25 points, and each negative reply (No) was assigned 0 points. The total knowledge score was 100 for four Omicron-related knowledge questions.

A score more than 50 was considered "good", a score between 25–50 was considered "average", and a score below 25 was "poor". Fear was elaborated as least-most afraid, least afraid, moderate afraid, high afraid, and extremely afraid on 05 points Likert scale. Similarly, 14 questions were about perception, including 11 questions specifically about Omicron (B.1.1.529). Furthermore, 02 questions were related to fear of Omicron (B.1.1.529).

### Statistical analysis

The descriptive analysis was presented as frequencies (N), percentages (%), and mean ± standard deviation (m ± SD). The association among dependent and categorical independent variables was determined by using the Chi-square test (*X2*) and multiple linear regression analysis using IBM Statistics SPSS version 23.

### Bio-ethical approval

Institutional bioethical committee (IBC) certificate **ORIC/SU/1079** was obtained from Office of Research Innovation & Commercialization (ORIC), University of Sindh, Jamshoro, Pakistan.

## Results

### Demographic features of respondents

The demographic characteristics of 353 respondents from different countries worldwide were received, recorded, and analyzed. Of the total respondents, more than half (60.6%) were females. 43.6% population was among the age group 18–27 years. The minimum age was 18, and the maximum age was 70 years, respectively, with a mean ±SD of 31.53 ±10.3, IQR = 14 at 95% CI (30.4–32.6). Moreover, 53.5% of respondents were single. More than half of the total respondents (53.3%) were postgraduates.

The majority of respondents (43.9%) were from Indonesia. Besides, 22.1% were from Malaysia, 15.3% were from Pakistan, 7.1% were from the Kingdom of Saudi Arabia, and 11.6% were from other countries of the world. **Table 1** highlights all the demographic features of the respondents.

**Table 1:** Demographic features of respondents.

Characteristics	N	%
**Gender**		
Female	214	60.6
Male	139	39.4
**Age Groups** 31.53±10.3		
18–27	154	43.6
28–37	119	33.7
38–47	54	15.3
48–57	16	4.5
58–67	6	1.7
68 and onwards	4	1.1
**Marital Status**		
Single	189	53.5
Married	160	45.3
Divorced/Widowed	4	1.2
**Country**		
Indonesia	155	43.9
Malaysia	78	22.1
Pakistan	54	15.3
KSA	25	7.1
Other Countries*	41	11.6
**Academic Qualification**		
Primary	19	5.4
Secondary	11	3.1
Graduation	135	38.2
Post-Graduation	188	53.3

Other Countries* = Australia, Canada, USA, Hungary, Egypt, India, Iraq, Jordan, Nigeria, Philippines, Qatar, UAE, Thailand, Yemen.

### COVID-19, vaccination & travel related information

**Table 2** elucidates that 80.7% of respondents never got infected with COVID-19. Moreover, 97.5% were vaccinated. Among the vaccinated population, 45.9% of respondents had completed their vaccination 3–6 months ago. Only 36.3% of respondents had received their booster dose of vaccine. Based on their travel history, 92.6% had not performed any journey in the last three months.

**Table 2:** Respondents history related to COVID-19 infection, vaccination and travelling.

Questions	N	%
**Did you ever get infected with COVID-19?**		
Yes	68	19.3
No	285	80.7
**Have you received COVID-19 vaccine?**		
Yes	344	97.5
No	9	2.5
**When did you complete your vaccination?**		
<3 months	108	30.6
3–6 months	162	45.9
>6 months	74	21.0
**Have you got the booster dose of vaccine?**		
Yes	128	36.3
No	225	63.7
**Have you travel lately in last 3 months to another country?**		
Yes	26	7.4
No	327	92.6

### Knowledge about COVID-19 & preventive measures

Based on the knowledge, 65.2% knew that COVID-19 vaccines help to enhance immunity. 89.8% of respondents knew that it is still essential to wear a face mask after being vaccinated. 89% of respondents knew that it is also crucial to sanitize hands every time, even after getting vaccinated. 73.7% knew that it is mandatory to refrain from public gatherings **(Table 3)**.

**Table 3:** Respondents’ knowledge regarding COVID-19 standard operating procedures (SOPs).

	Questions	Yes N (%)	No N (%)	Maybe N (%)
K1	Does COVID-19 vaccine help to boost immunity?	230 (65.2%)	10 (2.8%)	113 (32%)
K2	Is it still important to wear face mask after being vaccinated?	317 (89.8%)	15 (4.2%)	21 (5.9%)
K3	Is it important to sanitize hands?	314 (89%)	11 (3.1%)	28 (7.9%)
K4	Is it still important to refrain from public gatherings?	260 (73.7%)	32 (9.1%)	61 (17.3)

### Knowledge about Omicron (B.1.1.529) variant

**Table 4** represents the overall knowledge among the respondents about Omicron. The mean knowledge score about the Omicron variant was 3.18±1.14. It was noticed that 88.4% of people knew about the outbreak of Omicron, and 84.7% knew that the first case of Omicron was reported in South Africa. According to 70.8% of respondents, the spread of the Omicron variant is faster than the Delta variant. Similarly, 75.9% of respondents knew that the World health organization had declared Omicron (B.1.1.529) as a variant of concern (VOC) [26]. The majority (70%) of respondents knew that Omicron has many mutations.

**Table 4:** Respondents’ knowledge about Omicron (B.1.1.529) variant.

	Questions	Yes N (%)	No N (%)
K5	Do you know about outbreak of new variant named Omicron?	312 (88.4%)	41 (11.6%)
K6	Do you know that Omicron was first reported in South Africa?	299 (84.7%)	54 (14.3%)
K7	Do you know that Omicron has large number of mutations?	247 (70%)	106 (30%)
K8	Do you know that Omicron is declared as a variant of concern by W.H.O?	268 (75.9%)	85 (24.1%)

The knowledge of global respondents about each question regarding Omicron was analyzed by applying multivariate linear regression with association to various demographic variables such as gender, age, the country they belong and educational qualification level. The knowledge about outbreak of Omicron variant showed 0.8% variance, F (4, 348) = 1.68, adjusted R2 = 0.008, P = 0.15. The knowledge about the country of emergence of Omicron showed 5.8% variance, F = (4, 348) = 6.39, adjusted R2 = 0.058, P = 0.00. The knowledge about large number of mutations of Omicron, it showed negative 0.6% of variance, F = (4, 348) = 0.511, adjusted R2 = -0.006, P = 0.72. The knowledge about declaration of Omicron as variant of concern (VOC) (21), it showed negative1% variance, F (4, 348) = 0.103, adjusted R2 = - 0.010, P = 0.98. As shown in Table 4.1, that age was negative and significant in predicting the knowledge about the emergence of Omicron from South Africa. However, other factors were not significant in predicting the knowledge about evoke, emergence, mutation, and declaration of Omicron as a variant of concern. **Table 5** represents the knowledge among the global population about Omicron B.1.1.529 variant of COVID-19.

**Table 5:** Results of Multivariate linear regression analysis for knowledge about B.1.1.529.

**Variables**	**Outbreak of Omicron as a new variant (K5)**					**Omicron emerged from South Africa (K6)**				
	**B(95% CI)**	**S.E**	**Beta**	**T**	***p-value***	**B(95% CI)**	**S.E**	**Beta**	**t**	***p-value***
Constant	1.19 (0.98, 1.4)	0.10		11.2	0.00	1.289 (1.06, 1.5)	0.116		11.141	0.000
Gender	0.2 (-0.05, 0.93)	0.03	0.031	0.5	0.57	0.045 (-0.03, 0.12)	0.040	0.062	1.128	0.260
Age group	-0.34 (-0.06, 0.00)	0.01	-0.112	-1.9	0.49	-0.070 (-0.10, -0.03)	0.019	-0.204	-3.696	0.000
Country	-0.006 (-0.3, 0.02)	0.13	-0.026	-0.4	0.64	-0.022 (-0.051, 0.006)	0.015	-.085	-1.527	0.128
Education	-0.009 (-0.05, 0.03)	-0.023	-0.022	-0.3	0.7	-0.008 (-0.058, 0.042)	0.025	-.017	-0.300	0.764
**Variables**	**Mutations in Omicron (K7)**					**Omicron declared as VOC (K8)**				
	**B(95% CI)**	**S.E**	**Beta**	**T**	***p-value***	**B(95% CI)**	**S.E**	**Beta**	**t**	***p-value***
Constant	1.334 (1.03, 1.63)	0.152		8.76	0.000	1.192 (.912, 1.472)	0.142		8.381	0.000
Gender	0.029 (-0.075, 0.13)	0.053	.031	0.55	0.581	-0.002 (-0.100 0.095)	0.050	-0.003	-.046	0.964
Age group	-0.020 (-0.69, 0.029)	0.025	-.045	-0.79	0.425	-0.007 (-0.053 .039)	.023	-0.017	-0.305	0.760
Country	-0.009 (-0.047, 0.029)	0.019	-.028	-0.47	0.633	0.002 (-0.033 0.038)	0.018	0.007	0.120	0.905
Education	-0.007 (-0.7, 0.59)	0.034	-0.011	-0.19	0.846	0.018 (-0.044 0.080)	0.031	0.033	0.573	0.567

### Perception about other severe acute respiratory syndrome corona virus-2 variants

Nearly half (48.2%) respondents believe that COVID-19 is getting more dangerous day by day. However, 43.3% of respondents were unsure whether this is the most dangerous variant. Similarly, only 26.3% of respondents trust the COVID-19 vaccine to protect them from newly emerging variants **(Table 6)**.

**Table 6:** Respondents’ perception regarding COVID-19 and its variants.

	Questions	Yes N (%)	No N (%)
P1	Do you think that COVID-19 is getting more dangerous day by day?	218 (61.75%)	133 (38.25%)
P2	Do you think that Delta variant (Indian) was the most dangerous one?	202 (57.3%)	151 (42.7%)
P3	Do you think that available COVID-19 vaccines are enough to protect you from new SARS-COV2 variants?	167(47.3%)	186 (52.7)
P4	Do you think that the spread of Omicron is faster than Delta variant of COVID-19?	250 (70.8%)	103 (29.2%)
P5	Do you think that Omicron affects children more than adults?	159 (45%)	194 (55%)
P6	Do you think Omicron spread can be stopped by following COVID-19 SOPs and guidelines?	299 (84.7%)	54 (15.3%)
P7	Do you think Omicron can decline the global economic conditions?	285 (80.7%)	68 (19.3%)
P8	Do you think that spread of Omicron may lead to another nationwide lockdown?	263 (74.5%)	90 (25.5%)
P9	Do you think that spread of Omicron may increase the possibility of international restrictions for travelers?	308 (87.3%)	45 (12.7%)
P10	Do you think that Omicron can increase the risk of hospitalization?	280 (79.3%)	73 (20.7)
P11	Do you think that exposure to Omicron may be more fatal compared with other variants of COVID-19?	242 (68.6%)	111 (31.4%)
P12	Do you think that vaccination is the only way to cope with Omicron variant?	205 (58.1%)	148 (41.9%)
P13	Do you believe that people will take Omicron as a serious concern?	233 (66%)	120 (34%)
P14	Do you think that people around the globe have to adjust with far coming variants of COVID-19?	303 (85.8%)	50 (14.2%)

### Perception about Omicron (B.1.1.529) variant

Based on respondents’ perceptions, the majority (48.2%) believed that COVID-19 is getting more dangerous day by day. 43.3% of respondents have no clear perception that the Delta variant is the most dangerous among all Severe acute respiratory syndrome corona virus-2 variants, whereas 35.4% responded ’yes’. 70.8% perceived that Omicron is faster in spread than the Delta variant, 45% believed that Omicron affects children more, and 84.7% responded that the only way to stop the Omicron’s spread is by following standard operating procedures and guidelines that were announced for COVID-19 guidelines, for example, Social distancing, hygienic care and wearing a face mask [27,28].

Subsequently, 80.7% of people believed that Omicron would bring a global economic crisis, and 74.5% of respondents perceived that Omicron might bring lockdown scenarios to their countries. 87.3% of respondents think that international travel restrictions will be imposed. 79.3% of respondents think that Omicron can increase the risk of hospitalizations, and 58.1% believe that vaccination is the only way to cope with Omicron. 66% of respondents believe that people will take Omicron as a serious concern. Similarly, 85.8% of respondents thought the global population had to adjust to forthcoming COVID-19 variants.

### Perception regarding the fatality of Omicron (B.1.1.529) compared with previous variants

Based on the perception on a question (Do you think that exposure to Omicron may be more fatal compared with other variants of COVID-19?), there was no significant association of difference in perception among gender, age, and academic qualification. However, there was a significant association with difference in perceptions among various countries’ responses **(Table 7)**.

**Table 7:** Respondent’s perception regarding fatality of Omicron (B.1.1.529).

Variables	Perception		*X* ^*2*^	*p-value*
Gender	*Yes*	*No*		
Female	153	61	2.1	0.15
Male	89	50		
**Age Group**				
18–27	115	39	7.0	0.22
28–37	77	42		
38–47	31	23		
48–57	12	4		
58–67	4	2		
≥ 68	3	1		
**Country**				
Indonesia	107	48	9.2	0.05
Malaysia	62	16		
Pakistan	36	18		
KSA	14	11		
Others*	23	18		
**Academic Qualification**				
Primary	14	5	6.6	0.08
Secondary	10	1		
Graduation	99	36		
Post-Grad	119	69		

Other Countries* = Australia, Canada, USA, Hungary, Egypt, India, Iraq, Jordan, Nigeria, Philippines, Qatar, UAE, Thailand, Yemen.

Based on the perception (Do you think that exposure to Omicron may be fatal compared with other variants of COVID-19?) by applying multivariate linear regression with association to various demographic variables such as gender, age, and the country they belong and educational qualification level. Based on the perception about fatal nature of Omicron variant showed 1% variance, F (4, 348) = 1.8, adjusted R2 = 0.01, P = 0.13. **Table 8** illustrates the multivariable linear regression analysis of perception about Omicron.

**Table 8:** Multivariable linear regression about perception about B.1.1.529.

Variables	B(95% CI)	S.E	Beta	T	*p-value*
Constant	1.14 (0.848, 1.449)	0.153		7.509	.000
Gender	-0.041(-0.146, 0.064)	0.053	-.043	-.764	.445
Age	0.014 (-0.035, 0.063)	0.025	.032	.566	.572
Country	0.022 (-0.016, 0.061)	0.019	.066	1.153	.250
Education	0.046 (-0.021,0.112)	0.034	.078	1.358	.175

#### Fear in population towards Omicron (B.1.1.529)

Of the total 353 respondents, 73.9% (231) of respondents feared encountering Omicron, and only 26.1% (92) had no fear of the Omicron variant. The proportion of fear was more among females (79.9%) as compared to males (64.7%), *P < 0*.*05* and Malaysia (89.7%) as compared to Indonesia (77.4%), Pakistan (59.3%), Kingdom of Saudi Arabia (84%) and other countries (43.9%), *p-value*<*0*.*05*. However, no significant association between age group and qualification with fear was seen. See details in **Table 9.**

**Table 9:** Fear towardsB.1.1.529.

Variables	Yes N (%)	No N (%)	X^2^	*df*	*p-value*
**Gender**					
Male	90 (64.7%)	49 (35.3%)	10.04	1	0.002
Female	171 (79.9%)	43 (20.1%)			
**Age Group**					
18–27	116 (75.3%)	38 (24.7%)	2.74	5	0.73
28–37	86 (72.3%)	33 (27.7%)			
38–47	39 (72.2%)	15 (27.8%)			
48–57	11 (68.8%)	5 (31.3%)			
58–67	6 (100%)	0 (0%)			
68 and above	3 (75%)	1 (25%)			
**Country**					
Indonesia	120 (77.4%)	35 (22.6%)	37.6	4	0.000
Malaysia	70 (89.7%)	8 (10.3%)			
Pakistan	32 (59.3%)	22 (40.7%)			
KSA	21 (84%)	4 (16%)			
Others*	18 (43.9%)	23 (56.1%)			
**Qualification**					
Primary School	17 (89.5%)	2 (10.5%)	4.49	3	0.2
Secondary School	10 (90.9%)	1 (9.1%)			
Undergrad	99 (73.3%)	36 (26.7%)			
Post Graduate	135 (71.8%)	53 (28.2%)			

Based on the fear among respondents by applying multivariate linear regression with association to various demographic variables such as gender, age, and the country they belong and educational qualification level. Based on the perception about fatal nature of Omicron variant, showed 5.1% variance, F (4, 348) = 5.75, adjusted R^2^ = 0.051, *p-value < 0*.*05*. **Table 10** illustrates the multivariable linear regression analysis of fear towards OmicronB.1.1.529 variant.

**Table 10:** Multivariable linear regression analysis on fear towards B.1.1.529.

Variables	B(95% CI)	S.E	Beta	T	*p-value*
Constant	1.245 (0.96, 1.52)	0.142		8.796	0.000
Gender	-0.103 (-0.2, -0.003)	0.049	-0.115	-2.088	0.037
Age	-0.027 (-0.073, 0.01)	0.023	-0.064	-1.165	0.245
Country	0.056 (0.02, 0.09)	0.018	0.174	3.103	0.002
Education	0.032 (-0.02, 0.09)	0.031	0.058	1.037	0.301

### Extent of fear towards Omicron (B.1.1.529)

Based on the association among levels of fear and demographic variables, more females (36.9%) were extremely afraid as compared to males (22.3%), *p-value< 0*.*05*. Also, the majority of Malaysian respondents (42.3%) were more afraid of Omicron as compared to Indonesian (34.2%), Pakistani (31.5%), Saudi (16%) and other countries’ respondents’ *p-value<0*.*05* **(Table 11)**.

**Table 11:** Levels of fear towards B.1.1.529.

Variables	To what extent you are afraid of Omicron?					*X* ^*2*^	*p-value*
Gender	Least most	Least	Moderately	Highly	Extremely		
Female	8 (3.7%)	12(5.6%)	50 (23.4%)	65 (30.4%)	79 (36.9%)	13.7	0.008
Male	14 (10.1%)	14(10%)	34 (24.5%)	46 (33.1%)	31 (22.3%)		
**Age group**							
18–27	7 (4.5%)	11 (7.1%)	36 (23.4%)	45 (29.2%)	55 (35.7%)	11	0.94
28–37	7 (5.9%)	8 (6.7%)	28 (23.5%)	42 (35.3%)	34 (28.6%)		
38–47	6 (11.1%)	6 (11.1%)	14 (25.9%)	15 (27.8%)	13 (24.1%)		
48–57	2 (12.5%)	1 (6.3%)	4 (25%)	4 (25%)	5 (31.3%)		
58–67	0 (0%)	0 (0%)	1 (16%)	3 (50%)	2 (33.3%)		
≥ 68	0 (0%)	0 (0%)	1 (25%)	2 (50%)	1 (25%)		
**Country**							
Indonesia	3 (1.9%)	11(7.1%)	38 (24.5%)	50 (32.3%)	53(34.2%)	73.8	0.001
Malaysia	2 (2.6%)	1 (1.3%)	7 (9%)	35 (44.9%)	33 (42.3%)		
Pakistan	8 (14.8%)	4 (7.4%)	13 (24.1%)	12 (22.2%)	17 (31.5%)		
KSA	1 (4%)	5 (20%)	7 (28%)	8 (32%)	4 (16%)		
Others	8 (19.5%)	5 (12.2%)	19 (46.3%)	6 (14.6%)	3 (7.3%)		
**Academic Qualification**							
Primary	1 (5.3%)	0 (0%)	6 (31.6%)	7 (36.8%)	5 (26.9%)	14.2	0.20
Secondary	0 (0%)	0 (0%)	4 (36.4%)	3 (27.3%)	4 (36.4%)		
Graduation	5 (3.7%)	15 (11%)	26 (19.3%)	40 (29.6%)	49(36.3%)		
Post-Grad	16 (8.5%)	11 (5.9%)	48 (25.5%)	61 (32.4%)	52 (27.7%)		

## Discussion

The current research study was performed to evaluate the knowledge, perception, and level of fear against the new variant of COVID-19 (B.1.1.529) of COVID-19 among the global population. This assessment was essential for further enhancement of precautionary measures and aggressive vaccination drives against the COVID-19 in the global population as there is still a scarcity of definite treatment for COVID-19 in the world. In addition, the current scenario is still not favorable as people are not entirely adopting precautionary measures; therefore, the number of positive cases is inclining [29]. The outcomes of our research disclosed the vast number of demographic characteristics related to the level of knowledge, perception, and fear against the new variant (Omicron) of COVID-19. This study will further assist the healthcare authorities in enforcing standard operation procedures and vaccination drives against the pandemic. This cross-sectional study showed that the level of good knowledge regarding Omicron was 56.7%, suggesting a mediocre level of knowledge. Similar findings regarding the rate of knowledge have been reported from Pakistan [30] and China [31], where the level of knowledge accuracy about COVID-19 was 62.2% and 61.9%, respectively. A majority of respondents believed that despite being vaccinated, it is still necessary to wear a face mask (89.8%) and avoid public gatherings (73.7%) to limit the spread of Omicron. Consistent with our study, participants from the study of Muhammad et al. reported that face masking (75.7%) and social distancing (96.2%) are the essential precautionary measures to limit the spread of the COVID-19 pandemic [32]. Similar findings have been reported from Saudi Arabia [33] that face-masking (77.8%) and social distancing (78.0%) are even more critical than vaccination to prevent COVID-19 spread.

The public’s perception of Omicron is essential to understanding their practices. Our study shows that 68.6% of respondents believe that exposure to Omicron may be more fatal than other variants of COVID-19. Additionally, there was not any significant difference in perceptions among gender, age, and academic qualification regarding the fatality of Omicron with other variants of COVID-19. In comparison, other studies from Egypt [34] and Malaysia [35] showed comparatively higher percentages, 86.0%, and 79.0%, regarding the risk and dangerousness of previous variants of COVID-19.

The fear of the population towards the Omicron variant plays a vital role in following SOPs to curtail its spread. Our findings suggest that 73.9% of respondents feared the new Omicron variant. However, the level of fear was comparatively low (49.5%) towards the previous variant of COVID-19, as reported by Kumar et al. [30]. Moreover, the extreme level of fear towards Omicron was reported by 31.2% of respondents, lower than Muhammad et al., where 44.7% of health care workers were apprehensive about their health regarding COVID-19 [36]. Furthermore, the current research has also exposed an extreme level of fear among female respondents aged 18–27 years, residents of Malaysia, and graduates.

## Conclusion

As Omicron (B.1.1.529) is a new variant, there is still plenty of room to enlighten people about this infection. It is essential to spread clear knowledge about the effects and impacts of different variants of COVID-19, especially about B.1.1.529 variant, throughout the world. It is equally very essential to clear the mindset of the global population about the spread, extent of illness, and prevention from Omicron and far coming variants of COVID-19.

## Supporting information

S1 Data (XLSX)
